# Blood Glucose Monitoring and Sharing Amongst People With Diabetes and Their Facilitators: Cross-sectional Study of Methods and Practices

**DOI:** 10.2196/29178

**Published:** 2021-10-27

**Authors:** Amr Jamal, Shabana Tharkar, Weam Saleh Babaier, Shrooq Faisal Alsomali, Allulu Saad Alsulayhim, Monera Abdulkareem Alayuni, Nada Abdulaziz Aldakheel, Safa Sultan Al-Osaimi, Norah Alshehri, Mohammed Batais

**Affiliations:** 1 Evidence-Based Healthcare and Knowledge Translation Research Chair Department of Family and Community Medicine, College of Medicine King Saud University Riyadh Saudi Arabia; 2 Prince Sattam Chair for Epidemiology and Public Health Research Family and Community Medicine Department College of Medicine, King Saud University Riyadh Saudi Arabia; 3 College of Medicine, King Saud University Riyadh Saudi Arabia; 4 Department of Family and Community Medicine College of Medicine King Saud University Riyadh Saudi Arabia

**Keywords:** blood glucose monitoring, diabetes self- management, insulin users, Saudi Arabia

## Abstract

**Background:**

The last two decades have witnessed a burgeoning rise in the prevalence of diabetes globally. It has already reached epidemic proportions in Saudi Arabia, with reported high risk among women. As a result, diabetes monitoring and self-management programs are being highly prioritized for diabetes control and management.

**Objective:**

To investigate measuring and sharing practices of the self-monitoring of blood glucose (SMBG) among patients with type 1 or 2 diabetes using insulin.

**Methods:**

A cross-sectional study was conducted on a sample of 203 patients attending primary care clinics at a tertiary care center. The questionnaire assessed the measuring, recording, and sharing of SMBG practices of patients having diabetes with their physicians. The methods used for recording and sharing were categorized into paper-based and electronic-based. In addition, the determinants of the different methods used and frequency of sharing were analyzed.

**Results:**

The overall monitoring prevalence was 95% (193/203), and 57% (117/203) of participants shared the SMBG results. Among the 193 individuals that performed self-monitoring, 138 (72%) performed daily monitoring, and 147 (76%) recorded their blood sugar levels. Almost 55% (81/147) used paper-based materials like notebooks and paper for recording, while the rest (66/147, 45%) used digital devices like laptops and smartphones. A shift towards the use of digital devices and smart applications was observed in patients below 50 years of age. The digitally recorded blood glucose measurements were being shared thrice more often than the recordings made on paper or in notebooks (OR [odds ratio] 2.8; *P*=.01). Patients >50 years of age (OR 2.3; *P*=.02), with lesser formal education, married (OR 4.2; *P*<.001), with smaller family size (OR 2.6; *P*=.01), having type 2 diabetes (OR 4.1; *P<*.001) and any comorbid conditions (OR 2.6; *P*=.01) were associated with higher odds of using paper-based sharing methods. Only the female gender and type 2 diabetes were associated with increased frequency of sharing, while uncontrolled diabetes, the presence of other comorbidities, and duration of diabetes did not show any influence.

**Conclusions:**

Good monitoring and optimal sharing practices were found. Sharing using electronic devices can be emphasized. Diabetes self-management programs can incorporate the use of digital technology in training sessions. Digital literacy and its applications in health care may enhance SMBG practices resulting in better diabetes control.

## Introduction

Optimal glycemic control is central to the management of both type 1 and type 2 diabetes. Poor glycemic control has been causally associated with microvascular and macrovascular complications. Hence it is imperative to target and maintain optimum diabetes control [1-3]. Regular monitoring of blood glucose levels is an integral part of diabetes management [4]. Clinical monitoring of glycated hemoglobin levels (HbA_1c_) that determine the 3-month average blood glucose status and daily home monitoring of the capillary blood glucose levels called self-monitoring of blood glucose (SMBG) are the two principal methods of monitoring blood glucose levels [5]. According to the 2012 American Diabetes Association (ADA) guidelines, SMBG is recommended at least thrice daily for those on multiple insulin therapy and a minimum once daily for noninsulin users [6]. Diabetes management reached a significant milestone with the introduction of glucometer-based SMBG. Short-term benefits of regular monitoring of glucose levels include hypoglycemia prevention and the proven benefit to the physicians in adjusting the insulin doses. Scientific evidence suggests there is a substantial reduction in diabetes-related complications due to the long-term benefits of regular blood glucose monitoring [7-10].

Furthermore, in addition to monitoring, the practice of sharing blood glucose levels with the physicians is highly recommended by the consensus of organizations such as the ADA, International Diabetes Federation, and European Association for the Study of Diabetes in the holistic management of hyperglycemia [11-13]. However, the frequency of monitoring can be individualized according to the patient’s glycemic status, presence of other comorbidities and diabetes-related complications, lifestyle, and type of drugs administered [14]. Regular monitoring and sharing have been associated with significant predictors like motivation from the physician and family, fear of hypoglycemia, and the desire for good glycemic control [15]. Sharing SMBG results, in addition to HbA_1c_, has been the basis for drug dosing and physicians’ decision-making [14,15]. The frequency of SMBG monitoring and sharing influence the progressive monitoring behavior that ultimately has a profound impact on glycemic control. Technological advancements in monitoring devices have simplified the process of monitoring and sharing. Digital devices like smartphones, with specific health apps installations and glucometers linked to smart devices, offer a conducive medium for effortless and error-free sharing of measurements.

Scientific literature reporting SMBG practices among insulin users is often sparse in Saudi Arabia. Our study investigated the frequency of blood glucose monitoring and the methods adopted to measure, record, and share SMBG results by patients with diabetes and on treatment with insulin. We hypothesized that at least 50% shared the results with their physicians, and 50% of the patients used paper-based methods for recording and sharing. The associated factors that determine the sharing practices were also investigated. Additionally, the physicians’ advice on results-sharing and their perceptions on the adequacy of SMBG results in adjusting insulin dose was also determined. The results would provide a comprehensive understanding of the prevailing patient practices related to SMBG that may identify determinants of good monitoring and sharing practices. Moreover, the findings may suggest the improvement of diabetes education programs by adopting changing trends in digital technology use and facilitating patient empowerment in optimum diabetes management.

## Methods

### Study Design

A cross-sectional study design was incorporated to investigate the measurement of SMBG among patients with diabetes using insulin. The study was conducted from November 2019 until April 2020. Patients with a known diabetes diagnosis and on insulin treatment formed the primary sampling unit. The patients were identified from the appointment list on the hospital’s electronic health records at the primary care clinics, family medicine clinics, and specialized diabetes care centers of the university hospital. During the first 4 months of the study period, data was collected at the clinics, transitioning to telephone surveys during COVID-19 restrictions during March and April 2020. A well-trained team was involved in the data collection. Patients with cognitive impairment, pregnant women, and those requiring hospitalization were excluded. The selected patients’ physicians were also interviewed to assess the advice and use of shared SMBG results.

### Blood Glucose Measurements of Patients

Every patient with diabetes is usually provided with a glucose self-monitoring kit, including a diary, to maintain the self-management plan provided by the university hospital at the time of the first diagnosis or during the first follow-up visit. The patients record the SMBG results according to their preference and convenience using the given diaries or digital devices. Paper-based methods consisted of blood glucose readings measured and shared via diaries, notebooks, or paper, while smartphones, laptops, and glucometers were categorized as digital or electronic methods.

### Study Questionnaire and Sample Size

The questionnaire was comprised of two parts. The first section included questions regarding patient demographics, history of diabetes, and SMBG practices that were administered to the patients. The second part of the questionnaire included 4 questions were addressed to the patient’s attending physician, relating to the physician’s advice on sharing SMBG results and the use of results in adjusting the patient’s insulin dose. In addition, a pilot test was conducted on 20 subjects (excluded from the sample) attending primary care clinics to estimate the interview time, ensure comprehensibility, and test logistics. A sample size of 203 was obtained using the formula for a single proportion N=z^2^ x P x (1-P)/δ2, where *P*=75%, the proportion that shared the SMBG results during the pilot test, z=90% CI, and *P*=.05.

### Ethical Considerations

A consent form was attached to the questionnaire explaining the research purpose, research benefits, a statement of confidentiality, and a guarantee of participants’ right to drop out of the study at any stage. Participating in this study was nonobligatory, and no rewards were given to participants upon completing the questionnaire. Study approval was obtained by the department’s ethics committee (reference number CMED 305-F 14-2018-19).

### Data Analysis

Descriptive statistics such as mean and standard deviation were derived for continuous variables. Frequency and percentage were computed for binary and categorical data. Bivariate statistical analysis was carried out using appropriate statistical tests based on the type of study and outcome variables. Pearson’s chi-square test was used to test the differences in observed frequencies between the two groups, paper-based and electronic-based, considering the different categorical variables in 2 x 2 table. A *P* value of <.05 was used to report the statistical significance. The odds ratio (OR) and upper and lower 95% CI were taken from the risk estimate.

## Results

The final sample included 203 participants. The mean age of the study participants was 51.8 years (SD 16.6). Type 2 diabetes was predominant (155/203, 76%). The mean HbA_1c_ was 9.5%, and the majority (193/203, 95%) of the participants showed poor glycemic control (HbA_1c_ >7%). Table 1 illustrates the demographic characteristics of the study participants. Chronic diseases were reported in more than half (119/203, 58.6%) of the study participants. The most prevalent comorbidity was hypertension (90/203, 44.3%), followed by dyslipidemia (42/203, 20.7%). Other commonly reported comorbidities were thyroid disorders, mainly hypothyroidism, with asthma and kidney diseases accounting for 20% (40/203).

Table 2 displays the frequency of measuring, recording, and sharing of the SMBG results. A majority of participants (193/203, 95%) reported measuring their blood glucose levels. Almost 81% (156/197) measured themselves, and 19% (37/197) sought family assistance. Of the 193 individuals, 147 (76%) recorded the measurements, and among those who recorded, 117 (79%) shared the readings with their physicians. More than half of the participants (65/117, 55.6%) preferred paper-based methods like notebooks and paper sheets, while the rest (52/117, 44.4%) used digital devices like glucometers, mobile phones, laptops, and smartphone apps to share SMBG results. Significance testing with increased frequency of sharing showed only female participants and patients with type 2 diabetes were significantly associated with increased sharing (data not shown).

**Table 1:** Sociodemographic and clinical characteristics of patients with diabetes on insulin therapy.

Variable		Frequency (N=203)
**Age (in years), n (%)**		
	≤50	87 (42.9
	>50	116 (57.1)
**Gender, n (%)**		
	Female	109 (53.7)
	Male	94 (46.3)
**Nationality, n (%)**		
	Saudi	193 (95.1)
	Non-Saudi	10 (4.9)
**Education, n (%)**		
	School education	137 (67.5)
	Advanced education	66 (32.5)
**Employment status, n (%)**		
	Employed	55 (27.1)
	Retired	48 (23.6)
	Not employed	100 (49.3)
**Marital status, n (%)**		
	Married	150 (73.9)
	Not married	53 (26.1)
**Monthly family income, n (%)**		
	USD <2666	122 (60.1)
	USD >2667	81 (39.9)
**Family members, n (%)**		
	≤6	103 (50.7)
	>6	100 (49.3)
**Type of diabetes, n (%)**		
	Type 2	155 (76.4)
	Type 1	48 (23.6)
**Duration of diabetes (years), n (%)**		
	≤10	73 (36)
	>10	130 (64)
**HbA_1c^a^_** **, n (%)**		
	Uncontrolled (>7.0 %)	193 (95.1)
	Controlled	10 (4.9)
**Other chronic diseases**		
	Yes	119 (58.6)
	No	84 (41.4)
HbA_1c_, mean (SD)		9.5 (1.8)

^a^HbA_1c_: glycated hemoglobin levels.

**Table 2:** Frequency of self-monitoring of blood glucose practices.

Variables			Frequency (N=203)
Measuring, n (%)			193 (95.1)
Recording (n=193), n (%)			147 (76.2)
Sharing (n=147), n (%)			117 (79.6)
**# of times shared (n=117), n (%)**			
	Daily	25 (21.4)	
	Week	17 (14.5)	
	Monthly	36 (30.8)	
	Every 3 months	39 (33.3)	
**Methods of sharing (n=117), n (%)**			
	Notebook for recording blood glucose results	49 (41.9)	
	Paper	16 (13.7)	
	Glucometer	15 (12.8)	
	Laptop or Smartphone	20 (17.1)	
	Smart Applications	17 (14.5)	

Table 3 illustrates the determinants of the methods used for sharing. Patients aged >50 years were twice as likely to use paper-based methods (OR 2.3; *P*=.02) for recording and sharing the measurements. Being married increased the odds of using paper-based methods for sharing SMBG results by 4 times (OR 4.2; *P*<.001). Small family sizes (less than 6 family members) were also associated with the increased use of the paper-based methods (OR=2.6; *P*=.01). In addition, type 2 diabetes and the presence of any chronic ailment were associated with a greater likelihood of using paper methods. Although not reaching the level of statistical significance, those attaining formal education were twice as likely to rely on paper-based methods for recording and sharing the results with their physicians. Furthermore, digitally recorded blood glucose results were shared almost three times more frequently than those recorded on paper or notebooks (OR 2.8; *P*=.01). Other characteristics such as gender and HbA_1c_ were not significantly correlated with the methods of sharing.

Additionally, physicians’ role in patients’ SMBG practices was also analyzed. Most of the patients’ physicians (196/203, 96.6%) encouraged them to monitor their blood glucose levels regularly. In addition, almost 97.4% (114/117) of the physicians checked the SMBG results before adjusting the insulin dose. On the other hand, 60% (112/203) of the physicians perceived that SMBG measurements were adequate to adjust the patients’ insulin dose.

**Table 3:** Determinants of paper-based and electronic methods of sharing.

Variable		Paper-based sharing, n	Electronic-based sharing, n	χ^2^ value		*P* value		OR		95% CI	
**Age (years)**					4.86		*.02* ^a^		2.3		1.1-4.8
	>50	42	23								
	≤50	23	29								
**Gender**					1.17		.27		0.6		0.3- 1.3
	Male	26	26								
	Female	39	26								
**Education**					3.47		.06		2.0		0.9-4.4
	School education	47	29								
	Advanced education	18	23								
**Employment status**					0.00		.97		0.9		0.4- 2.3
	Not employed	32	27								
	Employed	18	15								
**Marital status**					11.77		*<.001*		4.2		1.8-9.8
	Married	54	28								
	Not married	11	24								
**Monthly family income (Saudi Riyal)**					0.17		.67		1.1		0.5-2.4
	USD <2666	40	30								
	USD >2667	25	22								
**Family members**					6.36		*.01*		2.6		1.2-5.5
	≤6	39	19								
	>6	26	33								
**Type of diabetes**					9.55		*<.002*		4.1		1.6-10.4
	Type 2	57	33								
	Type 1	8	19								
**Duration of diabetes (years)**					0.27		.59		1.2		0.5-2.6
	≤10	23	16								
	>10	42	36								
**HbA_1c_^b^**					–^c^		.23		0.2		0.1-1.6
	Controlled (<7)	2	5								
	Uncontrolled	63	47								
**Other chronic diseases**					6.12		*.01*		2.6		1.2-5.6
	Yes	47	26								
	No	18	26								
**Times they share**					6.24		*.01*		2.8		1.2- 6.4
	>1 Month	28	11								
	≤1 Month	37	41								

^a^*P* values in italic indicate differences between variables are statistically significant.

^b^HbA_1c_: glycated hemoglobin levels.

^c^Cell count less than 5 did not give the chi-square constant.

## Discussion

### Principal Findings

The measuring, recording, and sharing of SMBG practices in patients with diabetes using insulin were investigated. Some of the major findings of this study show that 95% (193/203 of the total study population monitored their blood glucose at any given time, and 72% (138/193) performed daily monitoring. Among those who monitored, close to 76% (147/193) recorded the measurements, of which 79% (117/147) shared it with their physicians. But the overall prevalence of sharing in the total sample was 57.6% (117/203) only. The majority of the participants (81/147, 55%) used notebooks or paper for recording SMGB readings, although smart applications were also frequently used. Being >50 years of age, with lesser formal education, married, with smaller family size, with type 2 diabetes, and the presence of comorbidities were significant determinants for using paper-based methods to share SMBG results. The majority of the physicians (196/203, 96.6%) constantly encouraged their patients to share the results, and most of them perceived that SMBG results are adequate for optimizing insulin doses.

Empirical evidence from studies worldwide suggests frequent monitoring of blood glucose to be significantly associated with effective glycemic control; hence, regular monitoring and sharing are highly recommended by the consensus [16-19]. Many international studies point towards a higher prevalence of daily blood glucose monitoring compared to sharing the results. For example, a nationwide Norwegian survey reported 70% of the patients practicing SMBG and less than 50% performing daily monitoring [20]. Another research from the United States noted 86% of insulin-users practice SMBG [21], while a regional study from Oman demonstrated a lower rate of 36%; nevertheless, all of them showed a lesser prevalence of results-sharing with physicians [22]. On the contrary, compared to these studies, our results reflect the higher prevalence of monitoring practices; however, sharing SMBG results with physicians was considered optimal.

Monitoring and sharing are two interlinked practices of diabetes self-management. Monitoring helps patients track their glycemic levels daily, plan nutritional and activity routines, and improve their quality of life, whereas sharing helps physicians optimize insulin doses. Physician’s motivation, in addition to diabetes self-management training and education programs, support the importance of monitoring and sharing practices. Our results showed that 93.6% (190/203) of the patients were encouraged by the physicians to monitor their glycemic status regularly, and 96.6% (196/203) were motivated to share the results. The encouraging attitude of the physicians reflected the high rate of monitoring; however, it did not effectively impact the rate of sharing. Of the 79% (117/147) who shared the SMBG results, 64% (75/117) shared once every month or more, and 21% (25/117) shared daily.

Further analysis showed an increased frequency of sharing to be associated with type 2 diabetes and being a woman. Although the presence of other comorbidities was not associated with increased sharing, patients in the “no comorbidity” group shared more often daily and weekly. Other variables like uncontrolled diabetes or longer duration of diabetes also did not influence sharing frequency. A similar regional study from Western Saudi Arabia demonstrated a prevalence rate of 70 % for measuring SMBG, with only 22% sharing the results with their physicians [23]. Our results showed higher rates of compliance when compared to the other regional literature. These are some important findings of our research which, if further investigated, might shed light on the causal reasons for frequent sharing that can be henceforth applied to augment effective diabetes management.

Furthermore, this study noted a slight preponderance towards the use of paper-based methods for sharing the results. However, the characteristics defining the method of use were unambiguous. Patients with higher age and those with lesser education preferred paper-based methods to digital devices. The other determinants like being married and the presence of comorbidities are also associated with age. One of the main reasons for the preponderance of paper-based methods could be related to the ease and comfort in recording the results right away. Since the patients are provided with a glucometer kit and notebook, many patients preferred saving the results in the notebook instantaneously compared to laptops or smartphones.

In the era of a digital revolution, the use of smartphones and digital devices is ubiquitous. One might expect a high dependency on smartphone applications for health-related information-sharing between patients and physicians. Our study has observed a transitional trend in the method of sharing, where the younger and more educated subjects preferred digital devices. Previous research has shown that uptake and sustained use of digital devices and applications for monitoring health depends on a number of factors like literacy, age, cognitive abilities, type and features of applications, and complexity of use [24]. Wildenbos et al [25] demonstrated poor usability and feasibility in using digital applications for health monitoring among older adults. Besides, complications in operations have largely contributed to aiding the discontinued use of digital applications for health benefits [26]. The findings from these studies establish the evidence in favor of predominant digital nonuse among senior people and the digitalization of younger patients, demonstrating consistency with our results. Identifying an emerging shift towards the use of digital technology in health care and contemplating the barriers of its application demonstrates the need to revise and reframe the structure of the standard diabetes education programs. Patient education programs could include training sessions demonstrating the use of digital devices and their applications in diabetes management.

Another key finding of the study is the adequacy of the shared blood glucose measurements in adjusting and customizing insulin doses. Almost 93% (188/203) of the physicians perceived shared results were adequate for optimizing the insulin dose. This is a major clinical use of SMBG. A recent review based on evidence from 26 studies found SMBG to be highly beneficial in titrating insulin doses. It has the potential to influence the physicians’ decision-making [27] as well as patients. Furthermore, some trials have demonstrated structured diabetes control programs achieving targeted glycemic control by using daily blood glucose monitoring results as a basis to self-adjust insulin dose in poorly or uncontrolled type 2 diabetes patients [28].

Finally, another interesting finding showed that those who used digital devices shared results three times more often than those who used paper methods. One of the reasons for using digital devices could be a relatively simpler and quicker process. Moreover, although not significant, the number of patients with uncontrolled diabetes in the device-using group was lesser than those in the paper-based group. In addition, the presence of comorbidities was significantly lesser in the device-using group. However, the role of confounders like age cannot be ruled out. Hence, digital devices can be considered as one of the facilitators of good sharing practices. Moreover, measurements from the digital devices can be synced, and the data can be stored in other devices for future reference. An Australian mobile health pilot program for diabetes control demonstrated digital device use and subsequent digital training to impact patients’ self-management of diabetes substantially [29]. With this additional evidence, we highly recommend prioritizing digital literacy in diabetes self-management training and education programs.

However, the study does contain certain limitations. Limited generalizability is one of the study's major limitations since the research was conducted at a single tertiary care government referral center. The smaller sample size is also a potential limitation.

### Conclusions

To our knowledge, this is the first study reporting the methods and determinants of the sharing practices of SMBG among insulin users in Saudi Arabia. The findings have good research and clinical implications. Future research exploring individual factors for patient preferences and monitoring adherence and detailed analyses of SMBG barriers are highly recommended. Furthermore, determinants, barriers, and facilitators of sharing and the effect of sharing on the disease status must be explored to understand the effective role of sharing SMBG results fully. However, this study provides resourceful literature and highlights the essentials of SMBG results sharing practices while recognizing the importance of the types of methods preferred that can substantially increase good practices. With diabetes beginning to affect more and more younger people, and owing to the widespread use of smartphones and other digital devices, digitalization can be considered one of the methods to increase diabetes monitoring. Numerous health applications in smartphones have been developed to assist in maintaining physical fitness, general health, and specific disease control like obesity and diabetes. Smartphone applications related to diabetes control and built-in glucometer software technologies enable users to keep track of their blood glucose and assist in optimum diabetes management through lifestyle modification strategies. Hence diabetes education and self-management programs can consider redesigning the curriculum to include training in the use of smartphone applications in diabetes self-management.

## Abbreviations

ADA: American Diabetes Association
OR: odds ratio
SMBG: self-monitoring of blood glucose
